# Microalgae as a Potential Functional Ingredient: Evaluation of the Phytochemical Profile, Antioxidant Activity and In-Vitro Enzymatic Inhibitory Effect of Different Species

**DOI:** 10.3390/molecules26247593

**Published:** 2021-12-15

**Authors:** Marta Vinha Vieira, Igor Piotr Turkiewicz, Karolina Tkacz, Claudio Fuentes-Grünewald, Lorenzo M. Pastrana, Pablo Fuciños, Aneta Wojdyło, Paulina Nowicka

**Affiliations:** 1 Department of Fruit, Vegetable and Nutraceutical Plant Technology, Faculty of Biotechnology and Food Science, Wrocław University of Environmental and Life Sciences, 37 Chełmonskiego Street, 51-630 Wrocław, Poland; marta.vieira@inl.int (M.V.V.); igor.turkiewicz@upwr.edu.pl (I.P.T.); karolina.tkacz@upwr.edu.pl (K.T.); 2 International Iberian Nanotechnology Laboratory, Food Processing and Nutrition Research Group, Av. Mestre José Veiga s/n, 4715-330 Braga, Portugal; lorenzo.pastrana@inl.int (L.M.P.); pablo.fucinos@inl.int (P.F.); 3 Bioscience Department, College of Science, Swansea University, Singleton Park, Swansea SA2 8PP, UK; c.fuentesgrunewald@swansea.ac.uk

**Keywords:** α-amylase, cholinesterase, pancreatic lipase, functional food, *Nannochloropsis oculata*, *Porphyridium purpureum*, *Arthorspira platensis*, *Chlorella vulgaris*

## Abstract

The functional food market has been in a state of constant expansion due to the increasing awareness of the impact of the diet on human health. In the search for new natural resources that could act as a functional ingredient for the food industry, microalgae represent a promising alternative, considering their high nutritional value and biosynthesis of numerous bioactive compounds with reported biological properties. In the present work, the phytochemical profile, antioxidant activity, and enzymatic inhibitory effect aiming at different metabolic disorders (Alzheimer’s disease, Type 2 diabetes, and obesity) were evaluated for the species *Porphyridium purpureum*, *Chlorella vulgaris*, *Arthorspira platensis*, and *Nannochloropsis oculata*. All the species presented bioactive diversity and important antioxidant activity, demonstrating the potential to be used as functional ingredients. Particularly, *P. purpureum* and *N. oculata* exhibited higher carotenoid and polyphenol content, which was reflected in their superior biological effects. Moreover, the species *P. purpureum* exhibited remarkable enzymatic inhibition for all the analyses.

## 1. Introduction

In recent decades, there has been an increasing awareness of the impact of the diet on human health. The concept of food, which was traditionally merely a means to satisfy hunger and meet metabolic requirements, has expanded to represent a potential way to prevent several nutrition-related diseases and improve physical and mental well-being [1,2]. According to the World Health Organization (WHO), dietary patterns and lifestyle habits constitute the main adjustable risk factors concerning the development of certain chronic diseases, such as obesity, type 2 diabetes, hypertension, among others, especially in developed countries [3]. Moreover, this new concept is particularly important in light of the rising cost of health care, the steady increase in life expectancy, and the desire to improve life quality [4].

As a result of such changes, the development of a new class of food known as functional food has arisen. Functional food may be defined as a natural or processed food that contains known biologically active compounds, which is able to provide benefits for the organism that are relevant either to one’s state of well-being and health; the management and reduction of diseases; or even physiological or psychological effects beyond the traditional nutritional role [5].

The creation of this new niche market has stimulated great interest in the investigation of natural resources, which could combine high nutritional value and functionality, to be used as a food ingredient in the development of novel functional foods. Among these, microalgae have been shown to be a valuable and sustainable viable alternative, as they represent a rich source of food-grade compounds and an almost unlimited field of exploration due to their abundant taxonomic diversity [6,7,8,9].

Microalgae are recognized for their interesting nutritional profile as it comprises not only a high protein content, but assorted carbohydrates, lipids, vitamins, and minerals. Moreover, these micro-organisms are capable of biosynthesizing several bioactive compounds, such as carotenoids, polyphenols, triterpenoids, polyunsaturated fatty acids, and polysaccharides [10]. It has been reported that the presence of this bioactive diversity has provided a significant range of biological properties to microalgae, which includes antioxidant, anti-inflammatory, anticancer, and antimicrobial effects, among others [11,12].

The application of microalgae for food fortification purposes has already been investigated for various products (e.g., cookies, bread, pasta, yogurt); nevertheless, our knowledge of the potential species to be used as a functional ingredients in the food industry and of their health-promoting benefits is still in its early years [13]. The Generally-Recognized-as-Safe (GRAS) species *Arthrospira platensis* and *Chlorella vulgaris* were the first ones to be commercialized as functional foods; therefore, they are among the most explored species in terms of the extraction and characterization of new bioactive compounds and possible biological properties [14]. On the other hand, a high number of species, such as *Nannochloropsis oculata* and *Porphyridium purpureum*, have been shown to be promising ingredients for the development of healthier food products, even though there is still much to discover about their functionality [15,16,17].

Considering the abovementioned findings, in this study we aimed at investigating the potential of the microalgae *P. purpureum*, *C. vulgaris*, *A. platensis*, and *N. oculata* for use as functional ingredients. For this purpose, we first evaluated the phytochemical profiles of the different species, i.e., polyphenols, carotenoids, chlorophylls, and triterpenoid content. In the second step, we focused on the assessment of the microalgae’s antioxidant capacity, as well as their in vitro health-promoting properties through enzymatic inhibition, i.e., acetylcholinesterase, α-amylase, and pancreatic lipase inhibitory effect. These assays are of particular importance nowadays due to the increasing number of patients with Alzheimer’s diseases, type 2 diabetes, and obesity. Accordingly, we hypothesized that these microalgae could represent a natural alternative for food fortification and the management of various chronic disorders.

## 2. Results and Discussion

### 2.1. Determination of Polyphenolic Content

Polyphenols constitute a large family of phytochemicals with great chemical diversity. Several biological properties have been widely associated with these compounds and studies suggest that their beneficial effects are mostly related to their antioxidant activity. The regular ingestion of polyphenols acts predominantly through the depletion of the oxidative stress caused by free radicals and through the maintenance of cellular redox homeostasis [18]. Therefore, the presence of such compounds is an important indicator of the functionality of microalgae biomass aimed at food fortification.

The polyphenolic content of the different microalgae species investigated in this study is displayed in Table 1. As can be observed, the species *C. vulgaris* presented the lowest amount of polyphenol compounds, differing significantly from the amount found in *P. purpureum*. The species *A. platensis* exhibited the most diversified polyphenolic content, with the detection of all three classes assessed. Moreover, more than half of the compounds of this species were composed of phenolic acids, whereas *P. purpureum*, *C. vulgaris*, and *N. oculata* were composed mostly or exclusively of flavan-3-ols.

Polyphenolic compounds are naturally present in microalgae and their content has already been investigated by some researchers. In Bhuvana et al. [19], for instance, the authors identified different phenolic acids and flavonoids by means of HPLC in methanolic extracts of *C. vulgaris* and *N. oculata*, such as chlorogenic acid, caffeic acid and luteolin 7-*O*-rutinoside for the former; and protocatechuic acid hexoside, quercetin pentosidehexoside and luteolin 7-*O*-glucoside for the latter. Similarly, da Silva et al. [20] quantified the polyphenolic content of ethanolic extracts of *A. platensis* obtained via different extraction methods through comparison with diverse phenolic acids and flavan-3-ol standards.

Different results can be achieved depending on the species and the extraction method/solvent used. Furthermore, the production of polyphenols in microalgae, as well as other bioactive compounds, is dependent on the environmental conditions and cultivation parameters, which ought to be considered before making a comparison of results between studies [21].

### 2.2. Determination of Carotenoid and Chlorophyll Content

Microalgae are recognized as an important source of natural pigments, such as chlorophylls and carotenoids, which have a fundamental role in cell photosynthetic metabolism. The consumption of products rich in these bioactive compounds, especially carotenoids, is associated with diverse health benefits, which has led to an increasing interest in the investigation and use of microalgae as a potential functional ingredient. In this study, an attempt at identifying chlorophyll and carotenoid compounds was first performed through analysis of the UV-Vis spectra of different standards (data not shown), followed by their quantification. The content of carotenoid and chlorophyll compounds for the different microalgae species can be found in Table 2. 

The chlorophyll content ranged from 0.29 g to 0.68 g per 100 g of freeze-dried biomass, with the lowest and highest amounts found in the species *P. purpureum* and *A. platensis*, respectively. Conversely, the carotenoid content presented the opposite trend for those two species, with the amount in the microalga *P. purpureum* being around 10-fold higher than the one found in *A. platensis*.

The microalga *A. platensis* exhibited the lowest content of carotenoid compounds, with 1.16 g per 100 g of freeze-dried biomass, represented mostly by zeaxanthin. This is consistent with the literature due to the high content of phycobiliproteins in cyanobacteria and the low content of carotenoids compared to green microalgae, for instance. Moreover, it was reported that the main carotenoid compound of this species may vary between zeaxanthin and β-carotene, depending on the sample source [22]. Finally, in our study only chlorophyll *a* was identified, which is in accordance with previous works [22,23].

On the other hand, in the species *P. purpureum* it was possible to identify the presence of chlorophyll type *a*, pheophorbide *a*, and possible derivatives, whereas the carotenoids were represented mainly by zeaxanthin, β-carotene, β-cryptoxanthin, and derivatives. Similar findings were reported by Juin et al. [24] in the pigment identification and structural analysis of the metabolites of an ethanolic extract of *P. purpureum* through UPLC-MS. The authors identified seven pigments or derivatives—in addition to the five we have mentioned in this study, the chlorophylls chlorophyllide *a* and pheophytin *a* were also identified using a standard database.

The microalga *N. oculata* exhibited a significant amount of carotenoids; however, no specific compound was identified when comparing them to the investigated standards. According to Bhuvana et al. [19], the main pigments present in this species are violaxanthin and chlorophyll *a*. In our study, chlorophyll *a*, pheophytin *a*, and possible derivatives were detected for *N. oculata*; chlorophyll *b* was absent, as previously reported in the literature [19].

In contrast with the other species explored, the microalga *C. vulgaris* showed the carotenoid lutein and both chlorophylls (*a* and *b*) as main pigments. Additionally, α-carotene and β-carotene derivatives were also identified. Those pigments were already reported in the literature to be present in an ethanolic extract of this species when examined through HPLC [25]. The presence of lutein as the most abundant carotenoid in *C. vulgaris* extracts is not unexpected, as many Chlorophyceae species are known to contain higher lutein levels than other carotenoids. Lutein is less hydrophobic than certain carotenoids, such as β-carotene; therefore, it is likely that a more polar solvent such as ethanol or methanol 80%, which was used in our study, would solubilize and extract lutein more effectively than other carotenoids [25].

### 2.3. Determination of Triterpenoid Content

Among the group of phytochemicals, terpenes are extensively found in nature, comprising approximately 30,000 identified compounds. They are synthesized from two five-carbon building blocks (isoprene) and, based on the number of structural molecules of isoprenes, they can be divided into mono-, sesqui-, di-, sester-, tri-, tetra-, and polyterpenes [26]. Triterpenoids have recently emerged as a unique group of compounds with several biological activities, such as anti-inflammatory, hepatoprotective, antimicrobial, immunomodulatory and, above all, with cytostatic effects in diverse cancer cells [26,27].

Microalgae are recognized as a potential source of different bioactives; however, the assessment and quantification of different triterpenoids in those microorganisms are still scarce in the literature. Table 3 shows the content of twelve different triterpenoid compounds found in the four microalgae considered in this work. The amount of triterpenoids identified ranged between 8.24 mg and 185.82 mg per 100 g of freeze-dried biomass, with the lowest and highest values found for *C. vulgaris* and *A. platensis*, respectively. Nevertheless, only *A. platensis* presented a value that was significantly different from the other species.

To date, there are no studies in the literature that have investigated the content of specific triterpenoid compounds in the microalgae *P. purpureum*, *C. vulgaris*, *A. platensis*, and *N. oculata*. However, a few authors have already stated the importance of triterpenoid compounds due to their antioxidant potential, reinforcing that the presence of these constituents in microalgae extracts increases the quality and beneficial impact of their use in human nutrition and health [28,29].

### 2.4. Evaluation of the Antioxidant Potential

**Figure 1:**
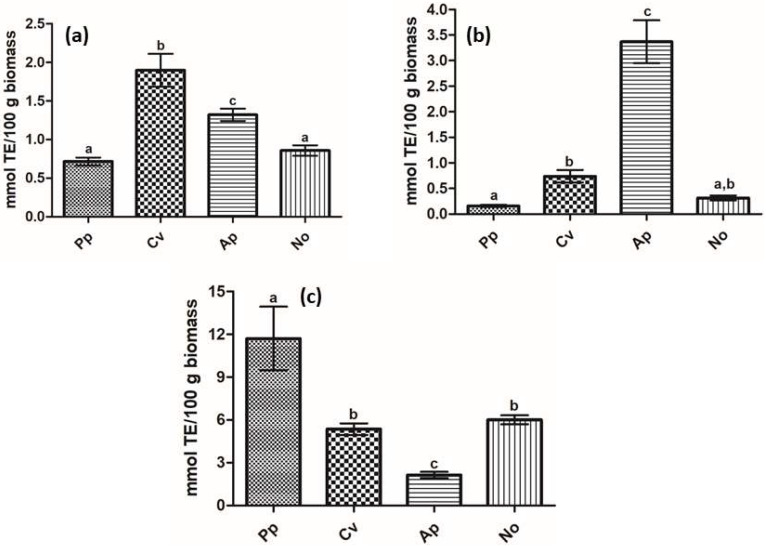
Antioxidant potential of the different microalgae species. (**a**) ABTS radical scavenging activity, (**b**) FRAP antioxidant assay, and (**c**) ORAC antioxidant assay. Different letters (a, b, c) indicate significant differences (*p* < 0.05). TE = Trolox equivalent. *Pp* = *P. purpureum*; *Cv* = *C. vulgaris*; *Ap* = *A. platensis*; *No* = *N. oculata*.

The antioxidant potential of the microalgae biomass was assessed through the ABTS, FRAP, and ORAC methodologies, and results can be found in Figure 1. It is noticeable that the species presented different trends depending on the method applied, which can be explained by their mechanisms of reaction, as well as by the properties of the antioxidant compounds present in the extracts, i.e., solubility, redox potential, specificity, and mechanism of action [30].

Both ABTS and FRAP assays are described to have a single electron transfer mechanism; however, the first is characterized by the reducing power of an antioxidant determined through its capacity to reduce a colored stable free radical (ABTS^•+^), whereas the second consists of the ability of antioxidant compounds to reduce Fe^3+^ ions to the blue Fe^2+^ ion complex [31,32]. ORAC analysis, on the other hand, has a mechanism based on the measurement of the inhibition of peroxyl radical-induced oxidations, hence reflecting classical radical chain-breaking antioxidant activity via hydrogen atom transfer [30].

Aside from the mechanism of reaction, another significant difference among these methods, which can be reflected in the antioxidant capacity of a sample, is the time of analysis. The ORAC assay measures the affinity of antioxidant compounds to neutralize the free radicals over a longer period of time (≥30 min), accounting for any potential lag phases in antioxidant activity, rather than providing a measurement of only fast-acting antioxidants. For the ABTS and FRAP assays, however, the neutralization of free radicals occurs at a particular point of time without accounting for slow-acting antioxidants [33].

In the ABTS radical scavenging activity, *C. vulgaris* exhibited the highest antioxidant activity, with a value of 1.90 mmol TE (Trolox Equivalent) per 100 g of biomass, whereas the lowest activities were found for *P. purpureum* and *N. oculata* (0.71 and 0.86 mmol TE/100 g, respectively). Similarly, the lowest activities were also found for those two species in regard to the ferric reducing power, determined using the FRAP method; nevertheless, in this case, the microalga *A. platensis* showed the highest antioxidant potential, with a value of 3.37 mmol TE/100 g. Lastly, a different result was observed for the ORAC assay, where *P. purpureum* displayed a significantly higher activity (11.70 mmol TE/100 g) and the lowest activity was found for *A. platensis* (2.14 mmol TE/100 g).

The antioxidant potential of microalgae has been reported to be highly dependent on bioactive diversity. Herein, we were able to identify positive correlations among the different bioactive content of the investigated microalgae species with the antioxidant activity results, demonstrated by Pearson’s correlation coefficient (*r*). The triterpenoid and chlorophyll content contributed significantly to the FRAP antioxidant activity, with *r* values of 0.973 and 0.931 for the former and latter, respectively. Those same bioactive groups also influenced the ABTS radical scavenging activity, although to a considerably lower extent (*r* = 0.241 and 0.140 for triterpenoids and chlorophyll compounds, respectively). Conversely, the ORAC value was strongly correlated with the presence of polyphenolic and carotenoid compounds (*r* = 0.682 and 0.875, respectively).

Overall, all microalgae species exhibited important antioxidant potential, comprising one or two radical scavenging mechanisms. This result was already expected due to the number of published studies proving the antioxidant capacity of microalgae and their compounds, thus suggesting that the investigated microalgae can be particularly interesting for food fortification purposes.

### 2.5. Assessment of the Cholinesterase, α-Amylase, and Pancreatic Lipase Enzymatic Inhibition Activities

In our study, we investigated the anticholinesterase activity of different microalgae to be used as a functional food ingredient for the prevention and management of Alzheimer’s disease (AD). This disease is an age-related neurodegenerative disorder, which occurs via complex pathophysiological mechanisms and is usually associated with memory loss and impairments in cognitive function. Although the etiology of AD has not yet been fully elucidated, previous reports have shown that several factors, such as oxidative stress, low levels of acetylcholine, β-amyloid aggregation, and the loss of synaptic neurons, may contribute to its development [34,35].

Current pharmacological approaches towards the treatment and management of AD comprise the use of cholinesterase inhibitors, which ensure adequate levels of acetylcholine at neurotransmission sites. Cholinesterase enzymes can be found in the brain mainly as acetylcholinesterase (AChE) and butyrylcholinesterase (BChE); however, the first is considered to be the key enzyme involved in acetylcholine hydrolysis and, consequently, in AD development [36]. Hence, it is hypothesized that acetylcholine levels are gradually lost during AD progression, but a delay in the loss of cognitive function can be achieved once the levels at the nerve synapse are restored. Moreover, cholinesterase inhibitors could also be used to avoid the formation of β-amyloidal plaques, which possess a crucial role in the prevention of neuronal death due to inflammation in AD [37].

As can be observed in Table 4, the AChE inhibitory potential of the microalgae ranged between 8.66% and 40.89%, with the lowest and highest values found for the species *A. platensis* and *P. purpureum*, respectively. Furthermore, the percentages for BChE inhibition were slightly lower compared to those for AChE for all the species, which could be explained by the structural differences between these enzymes and the interaction of the extract compounds on their active sites [38].

The cholinesterase inhibitory potential of the microalgae was strongly influenced by their content of polyphenolic and carotenoid compounds for both enzymes tested. For the AChE inhibitory activity, a Pearson’s correlation coefficients of 0.552 and 0.810 were found for the polyphenolic and carotenoid content, respectively. A similar trend was also observed for the BChE inhibitory activity, with *r* values of 0.568 and 0.809 for polyphenolic and carotenoids compounds, respectively. This result corroborates those of previous studies, which have evidenced that compounds of those bioactive groups may be potential cholinesterase inhibitors [39,40,41].

Assessments of the cholinesterase inhibitory effect of microalgae have already been performed by a few authors and the obtained results were highly dependent on the evaluated species, the extract concentration, and the extraction solvent used. Examples of microalgae that have shown significant AChE and BChE inhibition include the species *N. oculata*, *C. minutissima*, *Tetraselmis chuii*, *Rhodomonas salina*, *Botryococcus braunii*, *Chlorococcum* sp., and diverse strains of the genus *Nostoc* [37,42,43,44]. Regarding the microalgae of this study, the cholinesterase inhibition of aqueous protein extracts from *C. vulgaris* and *A. platensis* was evaluated in [45]. The authors reported an inhibitory effect against AChE of around 20% for *C. vulgaris* and 46% for *A. platensis*, which was proportionally associated with the protein amount found in each extract. The most promising microalga in our study in terms of AD management was the species *P. purpureum* and, to our knowledge, its anticholinesterase potential was herein evaluated for the first time.

Another important metabolic disorder, which is an emerging health concern worldwide, is type 2 diabetes (T2D). This disease is characterized by relative insulin deficiency caused by pancreatic β-cell dysfunction and insulin resistance in target organs. The increasing number of patients with T2D has been associated with the global growth in obesity, a sedentary lifestyle, and high energy intake [46].

The major source of glucose in our body comes from the hydrolysis of dietary carbohydrates. The pancreatic α-amylase and intestinal α-glucosidases enzymes are responsible for glucose generation via the diet. Therefore, it is suggested that the inhibition of these enzymes can be an important strategy for the management of T2D by acting in the reduction of the post-prandial increase of blood glucose [47,48]. In our study, we aimed to investigate the anti-diabetes activity of different microalgae through the assessment of their α-amylase inhibitory potential (Table 4). Results have shown IC_50_ values that are two- to four-fold lower for the species *P. purpureum* and *N. oculata* when compared to *C. vulgaris* and *A. platensis*. Particularly, *P. purpureum* exhibited a noteworthy activity, showing a reduction of 50% of the enzymatic activity with an extract concentration of 7.50 mg/mL.

As previously observed in relation to the anticholinesterase potential, the polyphenolic and carotenoid content of the microalgae deeply influenced their anti-diabetes activity (*r* = 0.921 and 0.999 for polyphenolic and carotenoid compounds, respectively). It has been reported that the hypoglycemic effect of polyphenolic compounds may be a result of their antioxidant potential, which is involved in the restoration of the insulin-secreting machinery in pancreatic cells, combined with the capacity to inhibit carbohydrates hydrolyzing enzymes, such as α-amylase [49]. The use of carotenoids in the prevention and control of T2D has also been widely described in the literature. Studies have revealed that there is an inverse association between the dietary intake of carotenoids and the risk of diabetes development, which can be explained by several mechanisms of action, such as enzymatic inhibition, the enhancement of insulin sensitivity, modulation of the immune system, and the prevention of oxidative stress [50,51].

There is a significant number of in vitro and in vivo studies demonstrating the anti-diabetes potential of the genera *Arthrospira* and *Chlorella*; however, the evaluation of their α-amylase inhibition is still scarce [52,53]. Hu et al. [54] have identified a peptide from *A. platensis* and observed significant α-amylase inhibition, with an IC_50_ value of 313.6 µg/mL. Recently, a study investigated the in vitro inhibitory activity of human salivary α-amylase of the pigment phycocyanin extracted from *A. platensis*, reporting an average of 51% inhibition [55]. In our study, those two species did not present a high inhibitory potential, possibly due to the difference in extract composition related to the cultivation parameters and solvent extraction.

Likewise, the anti-diabetes activity of the species *N. oculata* has already been reported, especially in cases of in vivo streptozotocin-induced diabetes [56,57]. In one of those studies, the authors observed severe expression of the enzyme α-amylase in the group with diabetes, which suffered a reduction after treatment with a microalga extract [56]. Conversely, the anti-diabetes potential of the microalga *P. purpureum* remains an unexplored field and its α-amylase inhibitory activity was evaluated in our work for the first time. Based on this result, *P. purpureum* seems to be a promising functional food ingredient; however, further studies should be conducted to understand more about the role of this species in diabetes management.

The use of digestive enzyme inhibition as a therapeutic approach is also particularly interesting for the management and treatment of the major risks associated with several chronic diseases. The incidence of diabetes mellitus, cardiovascular disorders, and cancer, among others, may be strongly related to obesity, which is characterized by dysregulated lipid homeostasis, with the excessive accumulation of fat and its inappropriate storage in the body [58,59].

Although caloric restrictions and exercise are known as the main pillars of action for obesity, one of the most important auxiliary strategies in the treatment of this metabolic disorder includes the administration of nutrient digestion and absorption inhibitors, in an attempt to reduce the energy intake through the gastrointestinal apparatus, without altering any central mechanisms. The pancreatic lipase is a key enzyme for triglyceride absorption in the small intestine, being responsible for the hydrolysis of 50%–70% of total dietary fats. Accordingly, the suppression of triglyceride absorption by lipase inhibition has been shown to be an interesting approach for obesity prevention [60,61].

Previous studies have already reported the lipase inhibitory potential of different microalgae biomass or compounds as a promising natural coadjutant tool in obesity management. In our work, we have reported anti-obesity activity, represented by the IC_50_ values, ranging from 3.26 to 23.24 mg/mL (Table 4), with the lowest and highest values found for *P. purpureum* and *A. platensis*, respectively. However, the lipase inhibitory activity of the species *N. oculata* did not differ significantly from the best result (IC_50_ = 3.38 mg/mL). Following the same tendency observed for the aforementioned enzymatic inhibition activities, the content of polyphenols and carotenoid compounds were immensely associated with the lipase inhibition found for the investigated microalgae, demonstrated by *r* values of 0.833 and 0.966, respectively.

The anti-obesity effect of *Chlorella* spp. has been previously investigated, and it was suggested that its possible mechanism of action could be due to the reduction of fat absorption in the intestinal tract. Furthermore, it was proposed that this reduction of blood lipid levels after the ingestion of *Chlorella* biomass could be related to the presence of specific compounds of this species, such as hydrophilic fibers, proteins, and β-carotene [62]. Concerning the pancreatic lipase inhibitory potential, Zhang et al. [63] have reported a good inhibitory effect (47.95%) for a novel decapeptide from *C. pyrenoidosa*, which was attributed to its hydrogen binding into catalytic sites of the pancreatic lipase. The inhibitory activity of the species *C. vulgaris*, on the other hand, had not previously been evaluated.

Several studies have demonstrated that *Arthrospira* spp. supplementation is particularly beneficial in the management of obesity. Some of the proposed mechanisms include anti-inflammatory and antioxidant properties, modifications in gut microbiota, the prevention of lipid accumulation, as well as pancreatic lipase inhibition [64,65]. In our findings, the microalga *A. platensis* exhibited a low level of enzymatic inhibition for all the evaluated biological properties, despite literature results suggesting otherwise. A possible explanation for this fact may be associated with the extract’s composition. Herein, we used a methanolic extract to perform the analyses; thus, many essentially hydrophilic compounds with significant health-promoting activities might not be present, such as the pigment phycocyanin.

Finally, there is a very limited number of studies reporting the anti-obesity potential of microalgae from the genus *Nannochloropsis*, whereas no studies were found aiming at the species *P. purpureum.* Based on our results, the microalgae *N. oculata* and *P. purpureum* showed remarkable anti-obesity activity, demonstrated by their pancreatic lipase inhibition; hence, these microalgae could represent a promising functional ingredient to support obesity management.

## 3. Material and Methods

### 3.1. Materials

The microalgae biomass of *P. purpureum*, *C. vulgaris*, *A. platensis*, and *N. oculata* were cultivated in the Department of Biosciences of Swansea University, freeze-dried (ScanVac Cool Safe, LaboGene; Lynge, Denmark), and stored at −20 °C until use. The microalgae cultivation methods of these species can be found [66]. All reagents used were purchased from Sigma-Aldrich (St. Louis, MO, USA). The standards of polyphenolic, carotenoid, chlorophyll, and triterpenoid compounds were bought from Extrasynthese (Genay, France).

### 3.2. Determination of Bioactive Content

#### 3.2.1. Determination of Polyphenolic Compounds

The quantification of polyphenolic compounds in the microalgae biomass was determined through Ultra-Performance Liquid Chromatography (UPLC), according to the method of Wojdyło et al. [67,68]. For the extraction, approximately 0.1 g of freeze-dried biomass was mixed with 5 mL of methanol:water:acetic acid:ascorbic acid (30:67:1:2, *v*/*v*/*v*/*w*) as the extracting solvent. This mixture was sonicated for 15 min in an ultrasonic bath (Sonic 6D, Polsonic; Warsaw, Poland), stored for 24 h at 4 °C, and sonicated again for an additional 15 min. Finally, the extract was centrifuged at 19,000× *g* for 7 min and filtered with a 0.2 µm hydrophilic membrane prior to analysis.

The analysis was performed on an Acquity UPLC system (Waters Corp., Milford; MA, USA) equipped with a photodiode detector (PDA). The chromatographic separation was carried out in reverse phase using an Acquity UPLC BEH C18 column (2.1 × 100 mm, 1.7 µm; Waters Corp., Milford, MA, USA), equilibrated at 30 °C. The mobile phase was composed of formic acid 4.5% (solvent A) and acetonitrile (solvent B) and samples were eluted according to the following linear gradient: 0–12 min, 1% to 25% B; 12–12.5 min, 100% B; 12.5–13.5 min, 1% B.

Detection was achieved at the wavelengths of 280 nm for flavan-3-ols, 320 nm for phenolic acids, and 360 nm for flavonols, with UV/Vis spectra monitored in the range of 200 to 600 nm. Quantification was achieved via the injection of known concentrations ranging between 0.05 and 5 mg/mL (r^2^ ≤ 0.9998) of (-)-epicatechin, chlorogenic acid, and quercetin-3-*O*-rutinoside, as representative standards of the flavan-3-ols, phenolic acids, and flavonol classes, respectively. All measurements were carried out in triplicate and the results were expressed as mg per 100 g of dried biomass.

#### 3.2.2. Determination of Carotenoids and Chlorophyll Content

The quantification of carotenoid and chlorophyll compounds in the microalgae biomass was also determined using Ultra-Performance Liquid Chromatography (UPLC), but according to the method of Wojdyło et al. [69]. For the extraction, approximately 0.1 g of freeze-dried biomass containing 10% of MgCO_3_ was shaken with 5 mL of a mixture consisting of methanol:acetone:hexane (1:1:2, *v*/*v*/*v*) in an orbital shaker (DOS-10L Digital Orbital Shaker, Elmi Ltd., Riga, Latvia) for 30 min in the dark. The supernatant was recovered through centrifugation at 19,000× *g* for 7 min. This extraction process was repeated two more times and the combined supernatants were evaporated to dryness under nitrogen (XCV–5400 XcelVap^®^ Evaporation System, Horizon Technology, Inc., Salem, NC, USA). Lastly, the resulting dry extract was dissolved in 1 mL of UPLC-grade methanol and filtered through a hydrophilic membrane, prior to analysis.

The analysis was performed on the same equipment and column as described in Section 3.2.1. Detection was achieved at the wavelengths of 430 nm and 450 nm, for chlorophylls and carotenoids, respectively, with UV/Vis spectra monitored in the range of 200 to 700 nm. The retention times and spectra were compared to those of bioactive standards and quantification was performed based on the standard calibration curves of all-*trans*-β-carotene, α-carotene, all-*trans*-lutein, β-cryptoxanthin, zeaxanthin, chlorophyll *a* and *b*, and pheophorbide *a*. All measurements were carried out in triplicate and the results were expressed as g per 100 g of dried biomass.

#### 3.2.3. Determination of Triterpenoid Compounds

The extraction of triterpenoid compounds was performed with approximately 0.1 g of freeze-dried microalgae biomass, which was mixed initially with ethyl acetate:hexane (1:1, *v*/*v*). This mixture was sonicated for 15 min in an ultrasonic bath, stored for 24 h at 4 °C, and centrifuged to remove the supernatant. Subsequently, a second extraction was performed with a mixture of dichloromethane:chloroform (1:1, *v*/*v*), and the same procedure described above was followed. Finally, both supernatants were combined and evaporated to dryness under nitrogen. The final residue was dissolved in 1 mL of UPLC-grade methanol and filtered through a 0.2 µm membrane prior to analysis.

The quantification and identification of the compounds were performed on an Acquity UPLC system (Waters Corp., Milford, MA, USA) equipped with a photodiode detector (PDA), according to Wojdyło et al. [68]. The chromatographic separation was carried out in reverse phase using an Ultisil XB-PAH column (4.6 × 250 mm, 5 µm; Welch Materials, West Haven, CT, USA), equilibrated at 23 °C. The mobile phase consisted of acetonitrile:water (88:12, *v*/*v*) at a flow rate of 1.0 mL/min, and detection was achieved at the 210 nm wavelength.

### 3.3. Preparation of Microalgae Extracts

Briefly, the freeze-dried microalgae biomass (~0.1 g) was mixed with 7 mL of methanol:water:HCl (79:20:1, *v*/*v*/*v*) as the extracting solvent. This mixture was sonicated for 15 min in an ultrasonic bath, stored for 24 h at 4 °C, and sonicated again for an additional 15 min. Lastly, the extract was centrifuged at 19,000× *g* for 7 min and filtered using a 0.2 µm membrane prior to analysis. The extracts were prepared in triplicate and they were used for all the in vitro biological activity assays.

### 3.4. Evaluation of Antioxidant Activity

The ABTS, FRAP, and ORAC assays were conducted as previously described by Re et al. [31], Benzie et al. [32], and Ou et al. [70], respectively. For the ABTS assay, first, an ABTS^•+^ reagent was prepared by mixing solutions of ABTS and potassium persulfate (1:0.5) and allowing it to react for 12–16 h. Subsequently, this solution was diluted with Milli-Q water to obtain an absorbance of 0.700 ± 0.02 at 734 nm. Microalgae extracts and the ABTS^•+^ reagent were then mixed and, after 6 min of reaction, the absorption was measured in the above wavelength. The results were calculated using a Trolox calibration curve (r^2^ = 0.9950), with concentrations ranging from 0.100 to 0.900 mmol.

For the FRAP method, microalgae extracts were diluted with distilled water and mixed with the FRAP reagent (acetate buffer, 2,4,6-Tris(2-pyridyl)-s-triazine (TPTZ) in HCl 40 mmol and FeCl_3_ × 6H_2_O in a ratio of 10:1:1, *v*/*v*/*v*). After 10 min of reaction, the samples were measured at a wavelength of 593 nm. The results were calculated based on a Trolox calibration curve (r^2^ = 0.9899), with concentrations ranging from 0.050 to 0.900 mmol.

Finally, for the oxygen radical absorbance capacity (ORAC) assay, the microalgae extracts were diluted in phosphate buffer and were mixed with fluorescein solution in a 96-well microplate. A 2,2′-azobis(2-amidinopropane)dihydrochloride (AAPH) solution was added and the fluorescence was recorded every 5 min after the addition of AAPH (excitation wavelength 487 nm, emission wavelength of 528 nm) for 50 min, at 37 °C. A blank (Fluorescein + AAPH) prepared with phosphate buffer instead of the extracts was also analyzed and Trolox was used as a standard. Results were calculated based on the differences in areas under the fluorescein decay curve between the blank and the samples.

All results were performed in triplicate and were expressed as Trolox Equivalent Antioxidant Capacity (TEAC) in mmol/100 g of microalgae biomass.

### 3.5. Determination of Acetylcholinesterase and Butyrylcholinesterase Inhibition

The acetylcholinesterase (AChE) and butyrylcholinesterase (BChE) inhibitory activity of the microalgae extracts were determined using the method described by Wojdyło et al. [67,68], Nowicka et al. [36] and Tkacz et al. [38] with slight modifications. Both assays were performed in a 96-well microplate with the addition of microalgae extract diluted in Tris-HCl buffer; Tris-HCl BSA buffer; substrate (acetylcholine iodine or butyrylcholine chloride, for AChE and BChE inhibition, respectively); DTNB, and of the enzyme. Control wells comprising the same reagents, but with buffer instead of the microalgae extract, were also assessed to provide the basal (uninhibited) AChE or BChE activity. Measurements were recorded at 412 nm in a microplate reader (Synergy2, BioTek Instruments Inc.; Winooski, USA) before enzyme addition and after 5 min of incubation at 37 °C in the presence of the enzyme. The percentage inhibition (Inhib.%) was calculated as the percentage of the difference in measurements of the sample before and after enzyme addition over the control. All samples were assayed in triplicate.

### 3.6. Determination of the Inhibitory Effect on the Digestives Enzymes α-Amylase and Pancreatic Lipase

The anti-diabetes and anti-obesity potential of the microalgae extracts were assayed through the α-amylase and pancreatic lipase inhibitory effect, according to the procedure described by Nowicka et al. [36,49], Tkacz et al. [38] and Wojdyło et al. 67,68], with slight modifications. For the anti-diabetes activity, different concentrations of the microalgae extract diluted in 0.1 M phosphate buffer (or as blank), mixed with a potato starch solution in a 96-well microplate with α-amylase dissolved in the above buffer to start the enzyme reaction. This final mixture was incubated for 10 min at 37 °C and the reaction was stopped by the addition of HCl. Subsequently, I_2_ in potassium iodide (KI) was added, and the absorbance was read at 600 nm in a microplate reader.

For the anti-obesity activity, first, lipase from the porcine pancreas was sonicated for 15 min, and then centrifuged at 10,000 rpm for 5 min. The initial reaction mixture was composed of different concentrations of the microalgae extract diluted in Tris-HCl buffer (buffer as blank), Tris-HCl buffer, and lipase solution. Following 5 min of incubation at 37 °C, *p*-nitrophenyl acetate solution was added as the substrate or water as a control. This final mixture was incubated for an additional 15 min and the absorbance was measured at 400 nm.

The inhibition percentage was calculated as the difference in measurements of the sample with and without enzyme addition for α-amylase, and with and without substrate addition for the lipase assay, over the control. The inhibitory activity for both analyses was expressed through IC_50_ values, which represent the amount of extract in mg/mL required to reduce the enzyme activity by 50%. All samples were assessed in triplicate.

### 3.7. Statistical Analysis

The results were statistically evaluated via an analysis of variance (ANOVA), followed by Tukey’s post hoc test, using GraphPad Prism 5.0 software. Pearson’s correlation coefficients (*r*) were calculated using Microsoft Excel 2013. Differences were considered statistically significant for *p* < 0.05.

## 4. Conclusions

The evaluation of the phytochemical profiles of the different microalgae revealed a diversified bioactive content, comprising polyphenols, carotenoids, chlorophylls, and triterpenoids in different proportions for all the species. The microalgae *P. purpureum* and *N. oculata* were particularly rich in carotenoid compounds, whereas *A. platensis* exhibited a more pronounced triterpenoid content. In this study, we also found that all microalgae possessed antioxidant potential through different mechanisms of reaction and in vitro anti-aging, anti-diabetes, and anti-obesity properties. Among the species investigated, *P. purpureum*, followed by *N. oculata*, exhibited the best results in the assessment of enzymatic inhibitory activities aimed at different metabolic disorders. This result increases the novelty of this work, considering that it is the first time these health-promoting properties have been attributed to the species *P. purpureum.* Furthermore, we have demonstrated that the presence of bioactive compounds strongly influenced the biological activity results, mostly regarding the carotenoid and polyphenol content. Therefore, the four microalgae biomasses assayed herein have proven to be a promising functional ingredient for food fortification due to their bioactive diversity and assorted biological properties; however, further investigation should be performed to evaluate their bioavailability and in vivo potential, giving special consideration to the unexplored species *P. purpureum.*

## Figures and Tables

**Table 1:** Polyphenolic content of the different microalgae species, determined through UPLC/PDA.

Microalgae Species	Flavan-3-ols	Phenolic Acids	Flavonols	Total Phenolic Content
*P. purpureum*	207.3 ± 14.72 ^a^	nd	nd	207.30 ± 14.72 ^a^
*C. vulgaris*	114.32 ± 6.11 ^b,c^	nd	nd	114.32 ± 6.11 ^b^
*A. platensis*	49.65 ± 4.18 ^c^	91.49 ± 7.36 ^a^	1.7 ± 0.14	142.84 ± 11.63 ^a,b^
*N. oculata*	174.65 ± 44.54 ^a,b^	22.08 ± 5.73 ^b^	nd	196.72 ± 50.02 ^a,b^

Values are expressed as mean ± standard deviation (n = 3) in mg per 100 g of freeze-dried biomass. Different letters (^a,b,c^) indicate significant difference (*p* < 0.05). nd—not detected.

**Table 2:** Carotenoid and chlorophyll content of the different microalgae species, determined through UPLC/PDA.

Microalgae Species	Total Chlorophyll Content	Total Carotenoid Content
*P. purpureum*	0.29 ± 0.02 ^a^	11.35 ± 2.62 ^a^
*C. vulgaris*	0.39 ± 0.06 ^a,c^	2.03 ± 0.53 ^b^
*A. platensis*	0.68 ± 0.03 ^b^	1.16 ± 0.11 ^b^
*N. oculata*	0.45 ± 0.01 ^c^	8.60 ± 0.33 ^a^

Values are expressed as mean ± standard deviation (n = 3) in g per 100 g of freeze-dried biomass. Different letters (^a,b,c^) indicate significant difference (*p* < 0.05).

**Table 3:** Triterpenoid content in the different microalgae species, determined through UPLC/PDA.

Triterpenoids	*P. purpureum*	*C. vulgaris*	*A. platensis*	*N. oculata*
Tormentic Acid	nd	0.30 ± 0.00	4.46 ± 1.99	1.55 ± 0.08
Alphitolic Acid	4.34 ± 0.69	0.44 ± 0.03	30.16 ± 5.19	4.77 ± 2.19
Maslinic Acid	0.99 ± 0.17	0.47 ± 0.02	17.70 ± 1.70	1.69 ± 0.64
Pomolic Acid	1.21 ± 0.10	0.76 ± 0.05	6.12 ± 0.44	1.27 ± 0.40
Corosolic Acid	1.68 ± 0.01	0.35 ± 0.02	13.68 ± 4.32	2.08 ± 0.41
Betulinic Acid	0.58 ± 0.25	0.15 ± 0.01	2.27 ± 2.04	1.05 ± 0.05
Oleanolic Acid	1.12 ± 0.80	0.37 ± 0.03	40.94 ± 11.33	7.17 ± 0.48
Ursolic Acid	2.06 ± 1.81	0.51 ± 0.06	59.74 ± 8.56	8.18 ± 4.96
Betulin	0.15 ± 0.03	1.95 ± 0.09	1.71 ± 0.61	1.20 ± 0.81
Erythrodiol	0.04 ± 0.02	0.14 ± 0.00	4.14 ± 1.15	0.21 ± 0.01
α-Boswellic Acid	0.04 ± 0.00	2.81 ± 0.08	5.44 ± 3.04	0.17 ± 0.03
Uvaol	0.23 ± 0.05	0.27 ± 0.04	2.07 ± 0.84	0.35 ± 0.05
Total Content	12.04 ± 0.43 ^a^	8.24 ± 0.05 ^a^	185.81 ± 18.34 ^b^	29.69 ± 2.58 ^a^

Values are expressed as mean ± standard deviation (n = 3) in mg per 100 g of freeze-dried biomass. Different letters (^a,b^) indicate significant difference (*p* < 0.05). nd—not detected.

**Table 4:** Evaluation of biological activities through enzymatic inhibition displayed by the different microalgae species.

	Cholinesterase Inhibition (% Inhib.)		α-Amylase Inhibition (IC_50 mg/mL_)	Pancreatic Lipase Inhibition (IC_50 mg/mL_)
	AChE	BChE		
*P. purpureum*	40.89 ± 4.44 ^a^	31.68 ± 1.15 ^a^	7.50 ± 2.68 ^a^	3.26 ± 0.94 ^a^
*C. vulgaris*	29.03 ±3.33 ^b^	24.14 ± 3.00 ^b^	28.72 ± 8.30 ^b^	9.81 ± 1.37 ^b^
*A. platensis*	8.66 ± 0.75 ^c^	6.85 ± 1.56 ^c^	31.04 ± 5.29 ^b^	23.24 ± 1.15 ^c^
*N. oculata*	29.89 ± 2.26 ^b^	28.01 ±1.39 ^a,b^	12.69 ± 5.53 ^a,b^	3.38 ± 0.38 ^a^

Values are expressed as mean ± standard deviation (n = 3). Different letters (^a,b,c^) indicate significant differences (*p* < 0.05).

## Data Availability

Data is contained within the article.

## Sample Availability

Samples of the compounds are not available from the authors.
